# Organizational readiness for knowledge translation in chronic care: a review of theoretical components

**DOI:** 10.1186/1748-5908-8-138

**Published:** 2013-11-28

**Authors:** Randa Attieh, Marie-Pierre Gagnon, Carole A Estabrooks, France Légaré, Mathieu Ouimet, Geneviève Roch, El Kebir Ghandour, Jeremy Grimshaw

**Affiliations:** 1 Research Center of the Centre Hospitalier Universitaire de Québec, Hôpital St-François D’Assise, 45 rue Leclerc, Québec, (QC), Canada; 2 Faculty of Nursing, Université Laval, Québec, Canada; 3 Faculty of Nursing and School of Public Health, University of Alberta, Edmonton, Alberta, Canada; 4 Department of Family Medicine, Université Laval, Québec, Canada; 5 Department of Political Science, Université Laval, Québec, Canada; 6 Ottawa Hospital Research Institute, Ottawa, Canada; 7 Faculty of Medicine, University of Ottawa, Ottawa, Ontario, Canada

**Keywords:** Organizational readiness, Conceptual models, Frameworks, Knowledge translation, Chronic care, Healthcare system

## Abstract

**Background:**

With the persistent gaps between research and practice in healthcare systems, knowledge translation (KT) has gained significance and importance. Also, in most industrialized countries, there is an increasing emphasis on managing chronic health conditions with the best available evidence. Yet, organizations aiming to improve chronic care (CC) require an adequate level of organizational readiness (OR) for KT.

Objectives: The purpose of this study is to review and synthesize the existing evidence on conceptual models/frameworks of Organizational Readiness for Change (ORC) in healthcare as the basis for the development of a comprehensive framework of OR for KT in the context of CC.

**Data sources:**

We conducted a systematic review of the literature on OR for KT in CC using Pubmed, Embase, CINAHL, PsychINFO, Web of Sciences (SCI and SSCI), and others. Search terms included readiness; commitment and change; preparedness; willing to change; organization and administration; and health and social services.

Study selection: The search was limited to studies that had been published between the starting date of each bibliographic database (*e.g*., 1964 for PubMed) and November 1, 2012. Only papers that refer to a theory, a theoretical component from any framework or model on OR that were applicable to the healthcare domain were considered. We analyzed data using conceptual mapping.

Data extraction: Pairs of authors independently screened the published literature by reviewing their titles and abstracts. Then, the two same reviewers appraised the full text of each study independently.

**Results:**

Overall, we found and synthesized 10 theories, theoretical models and conceptual frameworks relevant to ORC in healthcare described in 38 publications. We identified five core concepts, namely organizational dynamics, change process, innovation readiness, institutional readiness, and personal readiness. We extracted 17 dimensions and 59 sub-dimensions related to these 5 concepts.

**Conclusion:**

Our findings provide a useful overview for researchers interested in ORC and aims to create a consensus on the core theoretical components of ORC in general and of OR for KT in CC in particular. However, more work is needed to define and validate the core elements of a framework that could help to assess OR for KT in CC.

## Background

Healthcare systems are constantly changing, sometimes in subtle ways but at other times in major or even disruptive ways, in response to public health policy, market necessities, and technological advances [1]. At the same time, there is increasing international interest in organizational change as a lever for health care improvement. In several industrialized countries, an emphasis is placed on managing chronic health conditions, given the increasing pressures that they exert on health care systems [2]. However, significant gaps remain between evidence and the current management of chronic conditions [3]. For instance, among Canadians suffering from heart disease, only 50% receive proven therapies on a regular basis [2]. While organizational context has been shown to influence research utilization in practice, health care organization members and structures still need to have a sufficient level of readiness in order to implement research-based knowledge [4,5]. According to Holt and Helfrich, readiness is defined as ‘the degree to which those involved are individually and collectively primed, motivated and technically capable of executing the change’ ([6], p.S50).

### Organizational readiness for change

Organizational Readiness for Change (ORC) is a comprehensive attitude influenced simultaneously by the nature of the change, the change process, the organization’s context, and the attributes of individuals [7]. Change management researchers have emphasized the importance of establishing ORC and have recommended various ways to prepare for change [8,9]. However, the scientific knowledge base is limited, and ORC has not been subject to much empirical study yet [10].

The implementation of research-based practices to improve chronic care in various clinical settings has been incomplete, highlighting the difficulty of translating scientific knowledge to the ‘real-life’ care context [2]. Organizational readiness is likely to facilitate knowledge translation (KT) in implementing changes. In their extensive review, Weiner *et al*. [11] examined how ORC has been defined as a critical precursor to the successful implementation of complex changes in healthcare settings and how it has been measured in health services and in other fields. Recently, health services researchers have begun to theorize about developing measures of ORC in order to assess it empirically [8].

### Knowledge translation to improve chronic care

Knowledge Translation (KT) is a dynamic, iterative and complex process comprising knowledge creation and knowledge application to improve health, provide more effective health services and products, and strengthen the healthcare system [12]. KT strategies, targeted at individual healthcare workers, are insufficient to change healthcare professionals’ performance [13] and to influence patient outcomes. For this reason, other elements, such as contextual or organizational factors, must be taken into consideration [14-16]. According to Weiner *et al*. [11], ORC constitutes an appropriate concept to operationalize so as to permit the assessment of organizational capacity to engage in important change in healthcare.

In summary, ORC is seen as a key overarching concept to assess organizational members’ collective motivation and capability to implement change. Given the lack of knowledge on the theoretical foundations of ORC, our purpose was to review and synthesize the existing evidence on conceptual models/frameworks of ORC as the basis for the development of a comprehensive framework of OR for KT in the context of chronic care.

## Methods

### Eligibility criteria

We retained articles that refer to a theory, a theoretical component from any framework or model that was empirically applied to the healthcare domain. We also considered purely theoretical papers on OR that were applicable to the healthcare domain, but we excluded editorials, commentaries, and checklists. We excluded articles if they did not refer specifically to OR, did not concern the healthcare domain, were not about theoretical components or frameworks, or were in languages other than English, Finnish, French, Portuguese, Spanish or Swedish (languages that team members speak). We extracted the following information from selected publications: country, year of publication, type of study, methodological approach, and participants.

### Search strategy

We conducted a comprehensive review of the literature on conceptual frameworks and theoretical models relevant to ORC in healthcare in order to identify the core concepts that could be operationalized to assess organizational readiness for knowledge translation. An information specialist developed the search strategy on PubMed and then translated it across the other databases. The search strategy included four categories of keywords: Readiness, Commitment and Change, Organization and Administration, and Health and Social Services (Additional file 1). We searched the following databases: Pubmed, Embase, CINAHL, PsychINFO, Web of Sciences (SCI and SSCI), Business Source Premier, ABI/Inform, and Sociological Abstracts.

### Screening

Pairs of authors independently screened the published literature by reviewing their titles and abstracts. Then, the two same reviewers appraised the full text of each study independently. We also planned resolving discrepancies between authors through discussion, or involving a third reviewer as arbitrary, if necessary. We retained articles published in all languages, as long as they had an abstract in the ones identified above. We limited our search to articles published between the starting date of the bibliographic database (*e.g*., 1964 for PubMed) and November 1, 2012, which explicitly referred to the healthcare domain, and which applied the concept of ORC or equivalent terms (preparedness, commitment, or willingness to change). Finally, a third reviewer checked all the excluded and included studies.

### Extraction and classification

In order to classify theories, theoretical frameworks and models, we used the following definitions. According to Bacharach, ‘a theory may be viewed as a system of constructs and variables in which the constructs are related to each other by propositions and the variables are related to each other by hypotheses’ ([17], p.498). According to Miles and Huberman, a conceptual framework is a visual or written product that explains, either graphically or in narrative form, the main things to be studied – the key factors, concepts, or variables – and the presumed relationships among them [18], whereas a model, as stated by Sabatier [19] and Ostrom [20], represents a specific situation, is narrower in scope, and more precise in its assumptions.

Three authors (GR, MPG and RA) extracted the concepts, dimensions and sub-dimensions from the included publications. We used the CmapTools software developed by the Institute for Human and Machine Cognition (IHMC) [21]. This permitted us to graphically represent the core elements that were retrieved from the publications, as well as their relationships with the other elements. We classified each item into the dimension to which it was most often related in the literature, although we acknowledge that some elements could be classified in more than one dimension. We did this in three steps as follows.

First, three authors (GR, MPG and RA) placed the elements extracted from the frameworks and models identified in the included publications on a map in the CmapTools software without making a distinction regarding their nature (concept, dimension, sub-dimension). Concepts were represented in a hierarchical fashion with the most inclusive, most general concepts at the top of the map and the more specific, less general concepts arranged below [22]. Then, in a brainstorming session, we identified which elements were higher level theorization (concepts). From the remaining elements, we distinguished dimensions and sub-dimensions, which represented second or third level theorization. Third, we sought relationships among the concepts, dimensions and sub-dimensions that were created. Fourth, we placed closely related dimensions and sub-dimensions near each other within the concept to which they related in the concept map [23].

## Results

**Figure 1:**
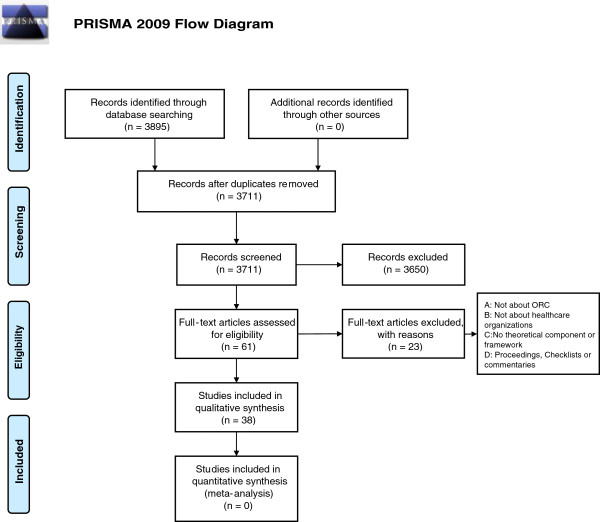
Study selection flow diagram.

The initial search strategy identified 3,711 references after duplicates were removed. After screening using the inclusion criteria, we retained 38 publications presenting 10 theories, theoretical models/components or conceptual frameworks of ORC (Figure 1). A total of 23 studies were excluded since they did not refer specifically to OR, did not concern the healthcare domain, or were not about theory, theoretical model or framework (Additional file 2).

### Characteristics of studies

In total, 6 (16%) of the 38 articles retained were published before 2000, 11 (29%) between 2000 and 2005, and 21 (55%) after 2005. A total of 28 (74%) of the studies took place in the USA, 4 (11%) in Canada, 5 (12%) in the UK, and 1 (3%) in Belgium. Study participants were: managers (24%), staff (19%), managers and staff (24%), not specified (3%), or not applicable (30%). Most of the studies (85%) were empirical, 5 (12%) were theoretical only and 1 (3%) was a review. A total of 19 studies (50%) had a quantitative research approach, and 7 (19%) had a qualitative one. In total, 5 studies (12%) used a mix of qualitative and quantitative methods. The remaining 7 studies (19%) were theoretical papers or reviews (Table 1).

**Table 1:** Characteristics of studies

		**Number (n** = **38)**	**% (100%)**
**Country of study**	USA	28	74
	Canada	4	11
	UK	5	12
	Belgium	1	3
**Year of publication**	Before 2000	6	16
	Between 2000-2005	11	29
	Since 2005	21	55
**Type of study**	Empirical	32	85
	Theoretical	5	12
	Review	1	3
**Research method**	Quantitative	19	50
	Qualitative	7	19
	Mixed	5	12
	None*	7	19
**Participants**	Managers	9	24
	Staff	7	19
	Both	9	24
	Not specified	2	3
	NA*	11	30

*:Theoretical studies and reviews.

### Organizational readiness theories, frameworks and models

We found 10 theories, theoretical models or conceptual frameworks of organizational readiness in healthcare that were published between 1998 and 2010, and these were explicitly presented in 29 (76%) publications. The 9 (24%) remaining publications applied social, contextual or other concepts pertaining to OR. They provided an application for readiness concepts, and they were considered as well in the concept map. They do not explicitly mention that they used a theoretical model or conceptual framework, but they present organizational readiness as a key component. In the following sections, we present the results (Table 2).

**Table 2:** Identified theoretical models and conceptual frameworks

**Theoretical models and conceptual frameworks (acronym)**	**Type of model/framework**	**Country of origin**	**Type of study**	**Setting**	**Year**	**Number of citation in studies**
1- Advancing Research & Clinical Practice through Close Collaboration (ARCC) [24]	Model	USA	Empirical	Clinical center	2002	2
2- A Comprehensive Measurement Model (CMM) [7]	Conceptual framework	USA	Empirical	Organizational change at the individual level	2007	2
3- A four category heuristic to conceptualize readiness for change [6]	Conceptual framework	USA	Theoretical	Health care organizations	2010	1
4- Diffusion of Innovations in Service Organizations (DISO) [25]	Model	UK	Empirical	Innovation in health service organizations	2004	1
5- Heuristic organizational information technology /systems innovation model (OITIM) [26]	Conceptual framework	USA	Empirical	Clinical IT/S innovation	2001	4
6- Practice Change Model (PCM) [27]	Conceptual framework	USA	Empirical	Primary care practice change	2004	4
7- Promoting Action on Research Implementation in Health Services (PARiHS) [28]	Conceptual framework	UK	Empirical	Implementation of practice in complex health care setting	1998	6
					2002	
8- Organizational Readiness for Change (ORC) [8]	Theory	USA	Theoretical	Health care services	2009	1
9- Readiness for Implementation Model (RIM) [29]	Model	USA	Empirical	Implementation of interactive health communication system	2010	2
10- Texas Christian University Program Change Model (TCU-PCM) [30]	Conceptual framework	USA	Empirical	Treatment program	2007	6

Eight of the theories, frameworks or models were developed in the United States and two in the United Kingdom. These theories, frameworks, or models conceptualized readiness empirically or theoretically from one of several perspectives, as suggested by Holt *et al*. [7], namely: change process, change content, change context, and individual attributes.

We retrieved one theory, the Organizational Readiness to Change theory (ORC), developed by Weiner [8], and nine theoretical models/conceptual frameworks.

The identified conceptual frameworks relevant to OR for KT are: Promoting Action on Research Implementation in Health Services (PARiHS), the Comprehensive Measurement Model (CMM), the Practice Change Model (PCM), the Texas Christian University Program Change Model (TCU-PCM), the Heuristic Organizational Information Technology/Systems Innovation Model (OITIM), and a framework called ‘A Four Category Heuristic to Conceptualize Readiness for Change.’ The models that proposed components relevant to OR for KT are: Readiness for Implementation Model (RIM), the Advancing Research & Clinical Practice through Close Collaboration (ARCC), and the Diffusion of Innovation in a Service Organization (DISO).

The PARiHS, TCU-PCM, OITIM and Practice CM models/conceptual frameworks appeared frequently (Table 2). The PARiHS framework was developed by Kitson *et al*. [28] in an attempt to represent the complexity of the change process involved in implementing various evidence-based interventions in healthcare [31].

The Texas Christian University Program Change Model (TCU-PCM) framework was developed by Simpson and Flynn [30] for planning and implementing innovations for the improvement of certain treatments.

The Heuristic Organizational Information Technology/systems Innovation Model (OITIM), a conceptual assessment framework developed by Snyder-Halpern [26], guides the decision-making processes of healthcare decision-makers in relation to organizational innovations.

The Practice Change Model (PCM) framework [27] was proposed by Cohen *et al*. for organizing, synthesizing, and understanding rationales for practice assessment approaches that ultimately inform the development of quality improvement interventions in primary care practices.

The Comprehensive Measurement Model (CMM), developed by Holt *et al*. [7] focuses on the pre-implementation stage, and is based on the concept of ability to succeed.

The Readiness for Implementation Model (RIM) by Wen *et al*. [29] was developed to help guide the implementation of interactive health communication systems (IHCS).

The purpose of the Advancing Research & Clinical Practice through Close Collaboration (ARCC) model, conceptualized originally by Melnyk [24], is to guide system-wide implementation of an intervention, in order to improve quality outcomes in healthcare organizations.

The Diffusion of Innovations in Service Organizations (DISO) model developed by Greenhalgh *et al*. [25] is used to organize insights concerning the adoption of an innovation in a service organization. Globally, it is envisioned as a memory aide for considering the different aspects of a complex situation and their interactions.

The broad conceptual framework, ‘A four category heuristic to conceptualize RC,’ proposed by Holt and Helfrich [6] ‘can facilitate thoughtful and meaningful reflection among leaders of health-care organizations regarding how to assess the readiness of their members and organization either qualitatively or quantitatively’ ([6], p.S52).

### Conceptual map data analysis

**Figure 2:**
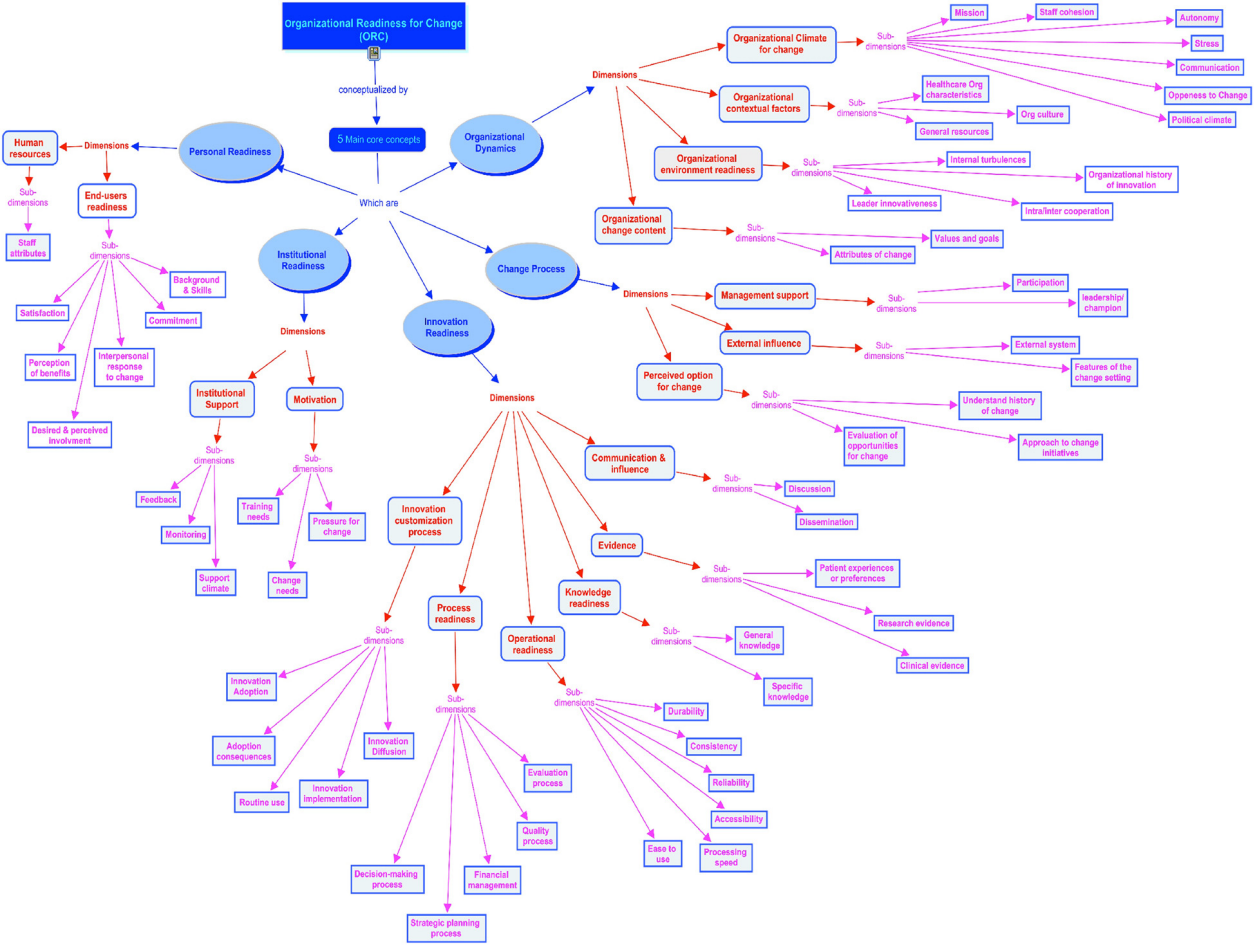
Concept map: synthesis of the theoretical findings of ORC components.

In the conceptual map (Figure 2), concepts (the highest level of theorization) are enclosed in circles. Concepts are linked by a connecting line to boxes representing dimensions (second level theorization), which, in turn, are linked to sub-dimensions (third level theorization). Words written on the lines define the relationship between the connected elements, thus helping us to organize and structure our thoughts to further understand information and discover new relationships [22,23].

After graphically analyzing the different components of OR gathered from the 10 theories, theoretical models and conceptual frameworks mentioned above, core concepts were identified as being common in the operationalization of OR for KT: Organizational Dynamics, Change Process, Innovation Readiness, Institutional Readiness, and Personal Readiness. Also, we identified 17 dimensions and 59 sub-dimensions related to these 5 concepts provided with their definitions in Additional file 3. A summary of the dimensions and sub-dimensions found in each theory, framework or model is presented in Additional file 4.

‘Organizational dynamics’ can support or suppress movement from one stage to another [32]. It refers to an appropriate organizational climate for change, contextual factors, environment readiness, as well as change content. The ‘change process’ concept refers to the steps to follow in order to implement change. It includes management support, external influence, as well as perceived options for change. ‘Innovation readiness,’ defined as the willingness and ability to adopt or implement an innovation in the workplace, refers to communication and influence, evidence, knowledge readiness, operational readiness, process readiness, and innovation customization process. ‘Institutional readiness’ includes motivation, values and goals readiness, as well as institutional support. The last concept, ‘personal readiness,’ encompasses human resources and end-users readiness. There were no discrepancies between authors.

## Discussion

We found and synthesized 10 theories, theoretical models, and conceptual frameworks of ORC in 38 publications as the basis for the development of a comprehensive framework of OR for KT in the context of chronic care. This review aimed to assess the current literature regarding the theorization and conceptualization of ORC in the healthcare domain in order to facilitate knowledge translation in implementing changes. This leads us to three main observations.

First, through the use of conceptual mapping, we have highlighted the relationships between the concepts, dimensions and sub-dimensions included in these models and frameworks. According to Watkins *et al*. [33], concept maps represent ideas or views from a large group of participants or stakeholders in an easy-to-interpret format. Concept maps visually illustrate relationships among words, concepts and facts. Moreover, concept mapping uses a structured process that can be replicated easily and reliably [33]. Understanding concepts and their underlying relationships is necessary to the acquisition of flexible, generalizable knowledge [22]. We propose a tentative map of the key components that underpin healthcare organizations’ readiness for KT, taking into consideration that a complete model of organizational change should embrace not only macro-level factors such as content, process and context, but also micro-level factors such the individuals involved [34]. Despite divergence in literature on ORC, the map helped us to draw the core concepts conceptualizing OR for KT and which were provided from the 10 proposed theories, theoretical models, or conceptual frameworks. We identified five core concepts, namely organizational dynamics, change process, innovation readiness, institutional readiness, and personal readiness. Some core concepts, dimensions, and sub-dimensions seem to be common to all of the proposed models but are described under distinct though equivalent terms, making it likely that an integrated framework on OR for KT in chronic care will be achievable. The concept of ‘organizational dynamics’ identified in our concept map, which was extracted from the Lehman model, is connected to common concepts or defined under equivalent terms in other theories, models or frameworks. For instance, a change is not likely to occur if staff cohesion, communication and openness to change *i.e*., organizational climate [32], or a good working relationship *i.e*., organizational climate [8], are not change oriented. Moreover, this conceptual map provides a synthesis of the information on the operationalization of the OR dimensions that could serve as the basis for the elaboration of an assessment tool to measure healthcare organizations’ readiness for KT in chronic care. The review and conceptual map provide a multidimensional and multi-level perspective on readiness for change, enabling a more complete picture of individual organizational readiness for change.

Second, the results of our review complement those of Weiner [10,11], finding little consistency with regard to terminology of concepts concerning readiness for change. Researchers have diverse ways of defining ORC. In our review, 19 publications (50%) used the term ‘readiness for change’ (35% in Weiner’s review), while others (50% in our review vs. 77% in Weiner’s review [11]) used some equivalent terms, including ‘capacity for change,’ ‘implementation readiness,’ ‘willingness, beliefs, state readiness/team readiness,’ and ‘innovation readiness.’ However, many authors would seem to refer to the same concept despite differences in the terms they use. For instance, Lehman *et al*. [32] consider ORC to include collective perceptions of ‘motivational readiness,’ ‘institutional resources,’ and ‘organizational climate.’ According to Weiner, ORC refers to organizational members’ motivation and capability to implement intentional organizational change [8]. Armenakis and colleagues used the term ‘readiness for change’ to indicate ‘organizational members’ beliefs, attitudes, and intentions regarding the extent to which changes are needed and the organization’s capacity to successfully make those changes’ ([35], p.681). In addition, our findings match those of Weiner [11], and Holt and Armenakis [7], and show the large amount of literature on the concept of readiness for change and its determinants. These reviews revealed the disagreements in current thinking and writing about the core concepts of ORC. To date, health services researchers investigating ORC are still independently examining specific types of organizational factors, using different theoretical perspectives to inform their research [11]. Unlike Weiner [11] and Holt and Armenakis [7], we did not limit our review to the history or definitions of readiness for change, but we focused on the application of this concept to the implementation of changes in healthcare organizations. Our review aimed to identify the similarities in the ORC models in order to conceptualize the core concepts that underpin organizational readiness for knowledge translation. This should help build a stronger theoretical base concerning ORC in general, and OR for implementing KT in chronic care in particular.

Third, the findings of the literature search showed that researchers have started only recently to propose theoretical frameworks and models of OR in the health domain. Reports of the application of these models and frameworks lack methodological detail on how to operationalize organizational readiness concepts. A strategy that could help address these limitations would be to begin to see the models/frameworks as being constituted by various theoretical positions, which some would view as strengths and others as weaknesses. Consideration also needs to be given to a framework's potential for development.

The ORC concepts, dimensions, and sub-dimensions identified in this systematic review will be further validated through an online Delphi study in preparation for the development of a comprehensive framework of OR for KT in the context of chronic care. In the next steps of this project, we will review existing measurement instruments, assess how they fit to the five core concepts identified, and develop a measurement instrument based on these five core concepts.

### Study limitations

This review has some limitations. First, it covers only articles published in peer-reviewed journals. Thus, the review might be subject to a publication bias if the gray literature contains conceptualizations of ORC that do not appear in peer-reviewed articles. Second, we focused only on the literature from the healthcare domain. ORC has been studied in other fields and relevant frameworks could be found from other disciplines, such as psychology, sociology, or management, for instance. However, given the relatively recent interest for ORC in healthcare, we chose to focus on theories and models that have been tested in this field and could thus be more easily applied to the study of OR for KT in chronic care. Third, we did not assess the quality of the included publications given the lack of a validated tool to assess the quality of theoretical studies. Finally, concept mapping includes only a high level representation of the performance area that is the subject of the concept. This method allows the representation of unidirectional and hierarchical relationships, and does not easily allow for the inclusion of detailed information about the complex relationships between dimensions and sub-dimensions. Using other graphical representations could be explored in order to depict the multiple influences that these constructs can share.

## Conclusion

Healthcare organizations need to be ready to adapt to changing demands and environments. ORC constitutes an appropriate concept to operationalize in order to assess organizational capacity to engage in implementing change in health care. The 10 theories, theoretical models and conceptual frameworks identified in the literature often have a narrow view of readiness, and skip one or several conceptual elements that are important for a comprehensive assessment of ORC. However, the conceptual map developed allowed us to identify the most relevant dimensions to consider for assessing OR for KT. This review and tentative conceptual map provide a useful overview for researchers interested in ORC and aims to create a consensus on the core theoretical components of ORC in general, and of OR for KT in chronic care in particular. However, more work is needed to define and validate the core elements of a framework that could help to assess OR for KT in the context of chronic care.

## Abbreviations

ARCC: Advancing research & clinical practice through close collaboration; CCM: Chronic care model; CMM: Comprehensive measurement model; DISO: Diffusion of innovations in service organizations; KTA: Knowledge-to-action; KT: Knowledge translation; OITIM: Heuristic organizational information technology/systems innovation model; ORC: Organizational readiness for change; OR: Organizational readiness; PARiHS: Promoting action on research implementation in health services; PCM: Practice change model; RIM: Readiness for implementation model; TCU-PCM: Texas Christian university program change model.

## Competing interests

The authors declare that they have no competing interests.

## Authors’ contributions

MPG, FL, CE, JG and MO conceived the idea and obtained funding. RA, MPG and EKG conducted the review. RA, MPG and GR conceived the conceptual map. RA and MPG wrote the first draft of the manuscript. All authors commented and contributed to the final manuscript.

## Supplementary Material

Additional file 1 Search strategy. Click here for file

Additional file 2 List of the excluded studies with the reason for exclusion. Click here for file

Additional file 3 Definition of concepts. Click here for file

Additional file 4 Dimensions and sub-dimensions found in 10 identified theoretical models and conceptual frameworks. Click here for file
