# Supplementary material for: Organizational readiness for knowledge translation in chronic care: a review of theoretical components
Source: Implement Sci. 2013 Nov 28;8:138. doi: 10.1186/1748-5908-8-138 (PMC4222028; doi:10.1186/1748-5908-8-138)
Supplement: Additional file 1 — Search strategy. [file 1748-5908-8-138-S1.pdf]

Search strategy

Pubmed

1- Readiness : Readiness[TIAB]
2- Commitment AND Change : (Commitment[TIAB] OR Preparedness[TIAB] OR Acceptance[TIAB] OR Willingness[TIAB]) AND (Change[TI] OR Changing[TI] OR Organizational Innovation[MH:NOEXP] OR Organizational Innovation*[TIAB] OR Organisational Innovation*[TIAB] OR Organizational change*[TIAB] OR Organisational change*[TIAB] OR Institutional change*[TIAB] OR Institutional innovation*[TIAB]) OR "Stages of change"[TIAB]
3- Organization and Administration : "Organization and Administration"[SH:NOEXP] OR Organizational Innovation[MH:NOEXP] OR Organisation*[TIAB] OR Organization*[TIAB] OR Institutional*[TIAB]
4- Health and social services : N/A
5- (#1 AND #2) AND #3

Embase

1- Readiness : Readiness:ti,ab
2- Commitment AND Change : ((commitment:ab,ti OR preparedness:ab,ti OR acceptance:ab,ti OR willingness:ab,ti) AND (change:ti OR changing:ti OR 'organizational innovation':ab,ti OR 'organizational innovations':ab,ti OR 'organisational innovation':ab,ti OR 'organisational innovations':ab,ti OR 'organizational change':ab,ti OR 'organizational changes':ab,ti OR 'organisational change':ab,ti OR 'organisational changes':ab,ti OR 'institutional change':ab,ti OR 'institutional changes':ab,ti OR 'institutional innovation':ab,ti OR 'institutional innovations':ab,ti)) OR 'stages of change':ab,ti
3- Organization and Administration : 'organization'/exp OR organisation*:ab,ti OR organization*:ab,ti OR institutional*:ab,ti
4- Health and social services : N/A
5- (#1 AND #2) AND #3 Limited to Embase

CINAHL (Ebsco)

1- Readiness : TI Readiness OR AB Readiness
2- Commitment AND Change : (TI (Commitment OR Preparedness OR Acceptance OR Willingness) OR AB (Commitment OR Preparedness OR Acceptance OR Willingness)) AND (TI Change OR TI Changing OR TI (Organizational Innovation* OR Organisational Innovation* OR Organizational change* OR Organisational change* OR Institutional change* OR Institutional innovation*) OR AB (Organizational Innovation* OR Organisational Innovation* OR Organizational change* OR Organisational change* OR Institutional change* OR Institutional innovation*) OR MH Organizational Change) OR TI "Stages of change" OR AB "Stages of change"
3- Organization and Administration : MH Organizational Change OR MH Organizations+ OR AB Organisation* OR TI Organisation* OR AB Organization* OR TI Organization* OR AB Institutional* OR TI Institutional*
4- Health and social services : N/A
5- (#1 AND #2) AND #3 Limited to "Peer Reviewed" Exclude Medline records

PsycINFO

1- Readiness : ti=readiness or ab=readiness
2- Commitment AND Change : ((ti=(Commitment OR Preparedness OR Acceptance OR Willingness) OR ab=(Commitment OR Preparedness OR Acceptance OR Willingness)) AND (ti=Change OR ti=Changing OR ti=("Organizational Innovation" OR "Organisational Innovation" OR "Organizational change" OR "Organisational change" OR "Institutional change" OR "Institutional innovation") OR ab=("Organizational Innovation" OR "Organisational Innovation" OR "Organizational change" OR "Organisational change" OR "Institutional change" OR "Institutional innovation") OR it="Organizational Change")) OR ti="Stages of change" OR ab="Stages of change"
3- Organization and Administration : it="Organizational Change" OR it=Organizations OR ti=(Organization* OR Organisation* OR Institutional*) OR ab=(Organization* OR Organisation* OR Institutional*)
4- Health and social services : N/A
5- (#1 AND #2) AND #3 Limited Peer-Reviewed Journals only

Web of science (SCI and SSCI)

1- Readiness : TS=Readiness
2- Commitment AND Change : TS=(Commitment OR Preparedness OR Acceptance OR Willingness) AND (TI= (Change OR Changing) OR TS=(("Organizational Innovation*") OR ("Organisational Innovation*") OR ("Organizational change*") OR ("Organisational change*") OR ("Institutional change*") OR ("Institutional innovation*"))) OR TS=("Stages of change")
3- Organization and Administration : TS=(Organization* OR Organisation* OR Institutional*)

4- Health and social services : TS=(Health* OR Medic* OR ("Social service"))
5- (#1 AND #2) AND #3 AND 4

Business Source Premier (EBSCO)

1- Readiness : TI Readiness OR AB Readiness
2- Commitment AND Change : (TI (Commitment OR Preparedness OR Acceptance OR Willingness) OR AB (Commitment OR Preparedness OR Acceptance OR Willingness)) AND (TI Change OR TI Changing OR TI (Organizational Innovation* OR Organisational Innovation* OR Organizational change* OR Organisational change* OR Institutional change* OR Institutional innovation*) OR AB (Organizational Innovation* OR Organisational Innovation* OR Organizational change* OR Organisational change* OR Institutional change* OR Institutional innovation*) OR DE "Organizational Change") OR TI "Stages of change" OR AB "Stages of change"
3- Organization and Administration : DE "ORGANIZATION" or DE "ORGANIZATIONAL change" OR AB (Organisation* OR Organization* OR Institutional*) OR TI (Organisation* OR Organization* OR Institutional*)
4- Health services and social : SU Health* OR TI Health* OR AB Health* OR SU Medic* OR TI Medic* OR AB Medic* OR DE "Social service" OR TI Social service* OR AB Social service*
5- (#1 AND #2) AND #3 AND 4 Limited to Scholarly (Peer Reviewed) Journals

Proquest ABI/Inform

1- Readiness : TI(Readiness) OR AB(Readiness)
2- Commitment AND Change : TI(Commitment OR Preparedness OR Acceptance OR Willingness) OR AB(Commitment OR Preparedness OR Acceptance OR Willingness) AND (TI (Change OR Changing OR "Organizational Innovation*" OR "Organisational Innovation*" OR "Organizational change*" OR "Organisational change*" OR "Institutional change*" OR "Institutional innovation*") OR AB("Organizational Innovation*" OR "Organisational Innovation*" OR "Organizational change*" OR "Organisational change*" OR "Institutional change*" OR "Institutional innovation*") OR SU("Organizational change")) OR TI "Stages of change" OR AB "Stages of change"
3- Organization and Administration : SU("Organizational change") OR SU(Organization) OR TI(Organisation* OR Organization* OR Institutional*) OR AB(Organisation* OR Organization* OR Institutional*)
4- Health and social services : SU(Health*) OR TI(Health*) OR AB(Health*) OR SU(Medic*) OR TI(Medic*) OR AB(Medic*) OR SU(Social services) OR TI(Social services) OR AB(Social services)
5- (#1 AND #2) AND #3 AND 4 Limited to "Peer Reviewed"

Sociological Abstracts database

1- Readiness : KW=Readiness
2- Commitment AND Change : KW=(Commitment OR Preparedness OR Acceptance OR Willingness) AND (TI= (Change OR Changing) OR KW=(("Organizational Innovation*") OR ("Organisational Innovation*") OR ("Organizational change*") OR ("Organisational change*") OR ("Institutional change*") OR ("Institutional innovation*"))) OR KW=("Stages of change")
3- Organization and Administration : KW=(Organization* OR Organisation* OR Institutional*)
4- Health and social services : KW=(Health* OR Medic* OR ("Social service"))
5- (#1 AND #2) AND #3 AND 4 Limited to "Peer Reviewed"
