# Supplementary material for: Organizational readiness for knowledge translation in chronic care: a review of theoretical components
Source: Implement Sci. 2013 Nov 28;8:138. doi: 10.1186/1748-5908-8-138 (PMC4222028; doi:10.1186/1748-5908-8-138)
Supplement: Additional file 2 — List of the excluded studies with the reason for exclusion. [file 1748-5908-8-138-S2.pdf]

List of the excluded studies with the reason for exclusion.

Author	Reason for exclusion
1- Zheng K. (2009) [1]	Not about ORC
2- Taylor W.A. (2004) [2]	Not about ORC
3- Jessup M.A. (2007) [3]	Not about ORC
4- Schurer J. (2010) [4]	Not about health care organizations
5- Larkey L. (1995) [5]	Not about ORC
6- Lin M.K. (2005) [6]	Not about ORC
7- McCarthy S.K. (2002) [7]	Not about ORC
8- Oser C.B. (2007) [8]	Not about ORC
9- Paddington S. (2002) [9]	Not about ORC
10- Raja S. (2008) [10]	Not about health care organizations
11- Rütten A. (2009) [11]	Not about health care organizations
12- Self D.R. (2009) [12]	Not about ORC
13- Walker H.J. (2007) [13]	Not about ORC
14- White D. (2000) [14]	Not about ORC
15- Whitten P. (2010) [15]	Not about ORC
16- Wisdom J. P. (2008) [16]	Not about ORC
17- Snyder-Halpern R. (1997) [17]	No theoretical model or conceptual framework
18- Prochaska J.M. (2001) [18]	Not about ORC
19- Khoja S. (2008) [19]	Not about ORC
20- Hebert M. (2004) [20]	Not about ORC
21- Dewan N. (2000) [21]	Not about health care organizations
22- Demiris G. (2004) [22]	Not about ORC
23- Hart P. (2008) [23]	Not about ORC

1. Zheng, K., et al., *Assessing organisational readiness for adopting an electronic health record systems: A case study in ambulatory practices.* Journal of Decision Systems, 2009. **18**(1): p. 117-140.
2. Taylor, W.A. and G.H. Wright, *Organizational Readiness for Successful Knowledge Sharing_Challenges for Public Sector Managers.* Information Resources Management Journal, 2004. **17**(2): p. 22.
3. Jessup, M.A., *Organizational change in a perinatal treatment setting: integration of clinical practice and policies on tobacco and smoking cessation.* J Psychoactive Drugs, 2007. **39**(4): p. 461-72.
4. Schurer, J., P. Kohl, and J. Bellamy, *Organizational Context and Readiness for Change: A Study of Community-Based Parenting Programs in One Midwestern City.* Administration in Social Work, 2010. **34**(2): p. 178.
5. Larkey, L. and C. Morrill, *Organizational commitment as symbolic process.* Western Journal of Communication, 1995. **59**(3): p. 193-213.
6. Lin, M.K., et al., *Motivation to change chronic illness care: results from a national evaluation of quality improvement collaboratives.* Health Care Manage Rev, 2005. **30**(2): p. 139-56.
7. McCarthy, S.K., *Availability of emergency contraceptive pills at university and college student health centers.* J Am Coll Health, 2002. **51**(1): p. 15-22.
8. Oser, C.B. and P.M. Roman, *Organizational-level predictors of adoption across time_Naltrexone in private substance-use disorders treatment centers.* Journal of Studies on Alcohol and Drugs, 2007. **68**(6): p. 852-861.
9. Paddington, S., M. Gilmartin, and D. Detmer, *Preparing to implement the NSF for Diabetes.* Diabetes & Primary Care, 2002. **4**(1): p. 13-18.
10. Raja, S., et al., *Success indicators for integrating mental health interventions with community-based rehabilitation projects.* Int J Rehabil Res, 2008. **31**(4): p. 284-92.
11. Rutten, A., et al., *Assessment of organizational readiness for health promotion policy implementation: test of a theoretical model.* Health Promot Int, 2009. **24**(3): p. 243-51.
12. Self, D.R. and M. Schraeder, *Enhancing the success of organizational change: Matching readiness strategies with sources of resistance.* Leadership & Organization Development Journal, 2009. **30**(2): p. 167-182.
13. Walker, H.J., A.A. Armenakis, and J.B. Bernerth, *Factors influencing organizational change efforts: An integrative investigation of change content, context, process and individual differences.* Journal of Organizational Change Management, 2007. **20**(6): p. 761-773.
14. White, D.B., *Instituting organizational learning for quality improvement through strategic planning nominal group processes.* J Healthc Qual, 2000. **22**(5): p. 13-8.
15. Whitten, P., B. Holtz, and L. Nguyen, *Keys to a successful and sustainable telemedicine program.* Int J Technol Assess Health Care, 2010. **26**(2): p. 211-6.

16. Wisdom, J.P., et al., *Preparing to implement medication algorithms: staff perspectives and system infrastructure.* J Psychiatr Pract, 2008. **14**(4): p. 209-15.
17. Snyder-Halpern, R., *Health services organizations computer innovation. Ready or not?* Comput Nurs, 1997. **15**(3): p. 147-52; quiz 153-4.
18. Prochaska, J.M., J.O. Prochaska, and D.A. Levesque, *A transtheoretical approach to changing organizations.* Adm Policy Ment Health, 2001. **28**(4): p. 247-61.
19. Khoja, S., R. Scott, and S. Gilani, *E-health readiness assessment: promoting "hope" in the health-care institutions of Pakistan.* World Hosp Health Serv, 2008. **44**(1): p. 36-8.
20. Hebert, M. and B. Korabek, *Stakeholder Readiness for Telehomecare: Implications for Implementation.* Telemedicine Journal and e-Health, 2004. **10**(1): p. 85-92.
21. Dewan, N.A., et al., *An internet-based survey of academic psychiatry and managed-care readiness.* Academic Psychiatry, 2000. **24**(2): p. 86-92.
22. Demiris, G., T.B. Partick, and S. Austin Boren, *Assessing patient safety awareness and needs in rural hospitals in one US state.* Informatics in Primary Care, 2004. **12**(3): p. 157-162.
23. Hart, P., et al., *Effectiveness of a computer-based educational program on nurses' knowledge, attitude, and skill level related to evidence-based practice.* Worldviews Evid Based Nurs, 2008. **5**(2): p. 75-84.
