# Supplementary material for: Organizational readiness for knowledge translation in chronic care: a review of theoretical components
Source: Implement Sci. 2013 Nov 28;8:138. doi: 10.1186/1748-5908-8-138 (PMC4222028; doi:10.1186/1748-5908-8-138)
Supplement: Additional file 3 — Definition of concepts. [file 1748-5908-8-138-S3.pdf]

## Definition of concepts

| Dimensions | Sub-dimensions |
|---|---|
| 1. Organizational climate for change: The collective appraisal of the internal organizational environment [1] | 1.1 Mission: Staff awareness of agency mission [1]<br><br>1.2 Staff cohesion: The focus on work group trust and cooperation [1]<br><br>1.3 Autonomy: Addresses the freedom and latitude staff members have in doing their work [2]<br><br>1.4 Stress: Pressure to do job [2]<br><br>1.5 Communication: Receptivity to suggestions from staff and the adequacy of information networks to keep everyone informed [1]<br><br>1.6 Openness to change: Management interest and efforts in keeping up with change [1]<br><br>1.7 Political change: Change across different organizational levels and areas [3] |
| 2. Organizational contextual factors: The circumstances under which the change is occurring [4] | 2.1 Healthcare organizational characteristics (structural): Factors that reflect the extent to which the circumstances under which the change is occurring enhance or inhibit the acceptance and implementation of change [5]<br><br>2.2 Organizational culture: values and beliefs of organizational members, which influence the character and quality of interpersonal relationships between members and between members and outsiders [6]<br><br>2.3 General resources: Qualities and characteristics that enable a practice to modify both its technical aspects and its values and/or beliefs regarding how it operates [7] |
| 3. Organizational environment readiness: State of the organization [8] | 3.1 Internal turbulences: Restructuring, staff turnover, miscellaneous organizational change [8]<br><br>3.2 Intra/Inter cooperation: Extent of cooperation between and within departments [8]<br><br>3.3 Organizational history of innovation: Prior history of successful innovation of practice implementation [8]<br><br>3.4 Leader innovativeness: Availability of a Leader of the innovation [8] |

| | |
|---|---|
| 4. Organizational change content: Refers to the particular change that is being introduced and its characteristics [4] | 4.1 Values and goals: Fit between existing health care organization structural and non-structural characteristics and innovation characteristics [3]<br><br>4.2 Attributes of change: Refers to 'what' factor of the change [4] |
| 5. Management support: Belief that organizational leaders are committed to change [4] | 5.1 Participation: The extent of leaders' participation in decision-making [7]<br><br>5.2 Leadership/ champion: Includes elements of teamwork, control, decision-making, effectiveness of organizational structures [6] |
| 6. External influence: Outside motivators used as rationale for or against change [7] | 6.1 External system: Health care affiliation or health care reimbursement [7]<br><br>6.2 Features of the change setting: Contextual features that can affect or be affected by the change [7] |
| 7. Perceived option for change: The extent to which practice members understand, evaluate, or reflect on opportunities for change [7] | 7.1 Understand history of change: What has this practice been through? What challenges has it faced in the past that gives shape to its current configuration? [7]<br><br>7.2 Approach to change initiatives: Practice members approach to change [7]<br><br>7.3 Evaluation of opportunities for change: Mechanisms used to review organizational change state [3] |
| 8. Communication & Influence: Communication through social networks of health professionals and interpersonal influence [9] | 8.1 Discussion: Development of shared meanings and values in relation to the change [9]<br><br>8.2 Dissemination: Planned, formal, often centralized strategies to spread the change [9] |
| 9. Evidence: The strength and nature of the evidence as perceived by multiple stakeholders [6] | 9.1 Patient experiences or preference: The proposed change should take into consideration the needs and preferences of patients [6]<br><br>9.2. Research evidence: The proposed change should be supported by published sources or participation in formal experiments [6]<br><br>9.3 Clinical evidence: The proposed change should be supported by clinical experience or professional knowledge [6] |
| 10. Knowledge readiness: Reflects both general and specific kinds of knowledge required by health care innovation decision makers [3] | 10.1 General knowledge: Previous healthcare organizations innovation patterns, decision-making processes, and innovation experiences [3]<br><br>10.2 Specific knowledge: Clinical practice standards, the impact of clinical practice standards on current practice processes and patient outcomes [3] |
| 11. Operational readiness: Fit between practice characteristics and operational features of existing practice [3] | 11.1 Durability: The ability of a practice to perform over a long period [10]<br><br>11.2 Consistency: The practice must fit actual patient care processes and work situations [10]<br><br>11.3 Reliability: The ability of a practice to perform its required functions under stated conditions for a specified period of time [10]<br><br>11.4 Accessibility: Practices must be available whenever users need them [10]<br><br>11.5 Processing speed: Practices must provide quick and value-added access to |

| | |
|---|---|
| | information [10]<br><br>11.6 Ease to use: Practices must be easy to use that they require little (or no) training [10] |
| 12. Process readiness: Fit between clinical innovation characteristics and existing practice processes and information support readiness [3] | 12.1 Evaluation process: Relates to how the organization measures its performance, and how feedback is provided to people within the organization [6]<br><br>12.2 Quality process: Process improvement recommendations [3]<br><br>12.3 Financial management: Budget management process used by the organization[3]<br><br>12.4 Strategic planning process: Type and role of individuals involved in development of the change strategic plan [3]<br><br>12.5 Decision-making process: Presence of decision-makers on key organizational committees (administrative and clinical) [3] |
| 13. Innovation customization process: Readiness assessment results are used to design the innovation to meet the needs of the health care setting [11] | 13.1 Innovation diffusion: The innovation is gradually introduced and diffused throughout the clinical practice environment [11]<br><br>13.2 Innovation implementation: The innovation is field tested as it is incrementally introduced into the clinical practice environment [11]<br><br>13.3 Routine use: Routinely utilization of the innovation in the clinical practice [11]<br><br>13.4 Adoption consequences: Innovation adoption creates consequences that can be either positive or negative for clinicians and the health care setting [11]<br><br>13.5 Innovation adoption: Usefulness and ease of use of the innovation [11] |
| 14. Motivation: Refers to the collective desire or interest to make an effort toward a particular goal [7] | 14.1 Pressure for change: From internal (e.g., staff) or external (e.g., regulatory and funding) sources. These pressures vary in intensity and form a summative index in which only at ''higher'' levels are they likely to reach sufficient threshold for a decision to take action [1]<br><br>14.2 Change needs: Valuation about strengths and weaknesses and issues that need attention [1]<br><br>14.3 Training needs: Perceptions of need for training in several general staff areas [1] |
| 15. Institutional support: Refers to how the organization sustains and supports change [12] | 15.1 Support climate: Support from senior leaders and middle managers of work environment, staff needs and funding support [5]<br><br>15.2 Monitoring: Incorporate rigorous monitoring of identified goals [9]<br><br>15.3 Feedback: Satisfactory feedback on change progress from those involved to improve processes [13] |
| 16. Human resources: Adequate human resources to implement the practice strategic plan [14] | 16.1 Staff attributes: Refers to professional growth, efficacy, influence, and adaptability that promote the change process [12] |
| 17. End-users readiness: Users characteristics | 17.1 Background and skills: Users past experiences or knowledge and skills level [3]<br><br>17.2 Commitment: Degree of ongoing commitment of those involved with the |

| | |
|---|---|
| and profile [3] | innovation [3]<br><br>17.3 Interpersonal responses to change: Desire for change[3]<br><br>17.4 Desired and perceived involvement: Degree of desired and perceived involvement in the innovation process [3]<br><br>17.5 Perceptions of benefits: The change is perceived to be personally beneficial [4]<br><br>17.6 Satisfaction: Users satisfaction with existing practice [3] |

**References**


1. Lehman, W.E., J.M. Greener, and D.D. Simpson, *Assessing organizational readiness for change.* J Subst Abuse Treat, 2002. **22**(4): p. 197-209.
2. Greener, J.M., et al., *Influence of organizational functioning on client engagement in treatment.* J Subst Abuse Treat, 2007. **33**(2): p. 139-47.
3. Snyder-Halpern, R., *Indicators of organizational readiness for clinical information technology/systems innovation: a Delphi study.* Int J Med Inform, 2001. **63**(3): p. 179-204.
4. Holt, D.T., et al., *Readiness for Organizational Change: The Systematic Development of a Scale.* Journal of Applied Behavioral Science, 2007. **43**(2): p. 232-255.
5. Holt, D.T., et al., *Are you ready? How health professionals can comprehensively conceptualize readiness for change.* J Gen Intern Med. **25 Suppl 1**: p. 50-5.
6. Helfrich, C.D., et al., *Organizational readiness to change assessment (ORCA): Development of an instrument based on the Promoting Action on Research in Health Services (PARIHS) framework.* Implement Sci, 2009. **4**: p. 38.
7. Cohen, D., et al., *A practice change model for quality improvement in primary care practice.* Journal of healthcare management / American College of Healthcare Executives, 2004. **49**(3): p. 155-68; discussion 169-70.
8. Wen, K.Y., et al., *Developing and validating a model to predict the success of an IHCS implementation: the Readiness for Implementation Model.* Journal of the American Medical Informatics Association : JAMIA, 2010. **17**(6): p. 707-13.
9. Greenhalgh, T., et al., *Diffusion of innovations in service organizations: systematic review and recommendations.* The Milbank quarterly, 2004. **82**(4): p. 581-629.
10. Snyder-Halpern, R., *Indicators of organizational readiness for clinical information technology/systems innovation: a Delphi study.* International journal of medical informatics, 2001. **63**(3): p. 179-204.

11. Snyder-Halpern, R., *Assessing health care setting readiness for point of care computerized clinical decision support system innovations.* Outcomes Manag Nurs Pract, 1999. **3**(3): p. 118-27.
12. Simpson, D.D., *A conceptual framework for transferring research to practice.* J Subst Abuse Treat, 2002. **22**(4): p. 171-82.
13. Simpson, D.D., *Organizational readiness for stage-based dynamics of innovation implementation.* Research on Social Work Practice, 2009. **19**(5): p. 541-551.
14. Jennett, P., et al., *Organizational readiness for telemedicine: implications for success and failure.* J Telemed Telecare, 2003. **9 Suppl 2**: p. S27-30.
