# Supplementary material for: Organizational readiness for knowledge translation in chronic care: a review of theoretical components
Source: Implement Sci. 2013 Nov 28;8:138. doi: 10.1186/1748-5908-8-138 (PMC4222028; doi:10.1186/1748-5908-8-138)
Supplement: Additional file 4 — Dimensions and sub-dimensions found in 10 identified theoretical models and conceptual frameworks. [file 1748-5908-8-138-S4.pdf]

Dimensions and sub-dimensions found in 10 identified theoretical models and conceptual frameworks.

**Theoretical models and conceptual frameworks (acronym)**	**Dimensions**	**Sub-dimensions**
1- Advancing Research & Clinical Practice through Close Collaboration (ARCC) [47]		Organizational culture
		Satisfaction
		Staff cohesion
2- A Comprehensive Measurement Model (CMM) [9]	Organizational contextual factors	Healthcare org characteristics
	Organizational change content	Values and goals
		Attributes of change
		Leadership/champion
		Participation
		Staff attributes
3- A four category heuristic to conceptualize readiness for change [8]		Organizational culture
		General resources
		Leadership/champion
		Support climate
		Background and skills
		Commitment
4- Diffusion of Innovations in Service Organizations (DISO) [49]	Communication and influence	General resources
	Motivation	Intra/inter cooperation
		Values and goals
		Leadership/champion
		Communication channels
		Discussion, Dissemination
		Evaluation process
		Decision-making process
		Commitment
		Political change
5- Heuristic organizational information technology /systems innovation model (OITIM) [39]	Management support	Healthcare org characteristics
	Knowledge readiness	Values and goals
	Operational readiness	General knowledge
	Innovation customization process	Specific knowledge
	Process readiness	Durability
	Human resources	Consistency
	End-users readiness	Reliability

		Accessibility
		Processing speed
		Ease to use
		Evaluation process
		Quality process
		Financial management
		Strategic planning process
		Decision-making process
		Innovation diffusion
		Innovation implementation
		Routine use
		Adoption consequences
		Innovation adoption
		Background and skills
		Interpersonal responses to change
		Commitment
		Desired and perceived involvement
		Perceptions of benefits
6- Practice Change Model (PCM) [41]	External influence	Staff cohesion
	Perceived option for change	Communication
	Motivation	Openness to change
	Institutional support	General resources
	Human resources	Participation
		External system
		Features of the change setting
		Approach to change initiatives
		Understand history of change
		Evaluation of opportunities for change
		Satisfaction
7- Promoting Action on Research Implementation in Health Services (PARiHS) [32]	Organizational contextual factors	Organizational culture
	Evidence	Leadership/champion
		Evaluation of opportunities for change
		Patient experiences or preferences
		Research evidence
		Clinical evidence
8- Organizational Readiness for Change (ORC) [10]		Organizational culture
	Organizational contextual factors	General resources

		Attributes of change
		Consistency
		Quality process
9- Readiness for Implementation Model (RIM) [46]	Motivation	Communication channels
	Organizational environment readiness	General resources
	Management support	Internal turbulences
	Communication and Influence	Intra/Inter cooperation
		Organizational history of innovation
		Leader innovativeness
		Leadership/champion
		Ease to use
		Quality process
		Training needs
10- Texas Christian University Program Change Model (TCU-PCM) [34]	Organizational climate for change	Mission
	Organizational contextual factors	Staff cohesion
	Management support	Autonomy
	Motivation	Stress
	Institutional support	Communication channels
		Openness to change
		General resources
		Pressure for change
		Change needs
		Training needs
		Monitoring
		Feedback
		Staff attributes
